# Gene Mutations Related to Glucocorticoid Resistance in Pediatric Acute Lymphoblastic Leukemia

**DOI:** 10.3389/fped.2022.831229

**Published:** 2022-06-06

**Authors:** JinFang Zhang, LingJi Zeng, YuLian Wang, JianWei Pan, XingDong Li, Bei Feng, Quan Yang

**Affiliations:** ^1^Department of Paediatric Hematology, Guangdong Provincial People's Hospital, Guangdong Academy of Medical Sciences, Guangzhou, China; ^2^Department of Hematology, Guangdong Provincial People's Hospital, Guangdong Academy of Medical Sciences, Guangzhou, China

**Keywords:** TTN, NOTCH1, gene mutation, acute lymphoblastic leukemia, drug resistance

## Abstract

**Objective:**

To investigate the correlation between gene mutations and glucocorticoid resistance in pediatric acute lymphoblastic leukemia (ALL).

**Methods:**

A total of 71 children with ALL admitted to our center between September 2019 and September 2021 were enrolled. DNA obtained from bone marrow or peripheral blood samples at initial diagnosis was used for genetic testing *via* whole exome sequencing. Meanwhile, patient clinical information was collected. Subsequently, the correlations of gene mutations with clinical features and glucocorticoid resistance were analyzed.

**Results:**

Of the 71 children enrolled, 61 (85.9%) had B-cell ALL (B-ALL) and 10 (14.1%) had T-cell ALL (T-ALL). The five genes with the highest mutation frequency in B-ALL were *TTN* (24.4%), *FLT3* (14.6%), *TP53* (14.6%), *MUC16* (9.8%), and *EPPK1* (9.8%). In contrast, those with the highest frequency in T-ALL were *NOTCH1* (54.5%), *FBXW7* (27.3%), *TTN* (27.3%), *MUC16* (27.3%), and *PHF6* (18.2%). Upon statistical analysis, *TTN* and *NOTCH1* mutations were found to be associated with prednisone resistance. Further, *TTN* and *MUC16* mutations were associated with a lower age at diagnosis, and *NOTCH1* mutations were associated with T-ALL in female patients. Leukocyte counts and LDH levels did not differ based on the presence of any common gene mutation, and no association between these gene mutations and overall survival was observed.

**Conclusions:**

Our study is the first to demonstrate the association between *TTN* mutation and glucocorticoid resistance in ALL. Our findings could guide strategies for overcoming drug resistance and aid in the development of drug targets.

## Introduction

Pediatric acute lymphoblastic leukemia (ALL) is the most common malignancy in children and adolescents, accounting for ~25% of tumors in children aged ≤15 years (1). Due to the development of combination chemotherapy, targeted therapy, cell therapy, and hematopoietic stem cell transplantation, the prognosis of pediatric ALL has improved considerably. The overall survival (OS) for pediatric ALL in individuals with standard risk is now more than 80%, and St. Jude Children's research hospital reported a 5-year OS of 93.5% in this group (2).

Nevertheless, there remain cases of ALL that cannot be cured. In these cases, refractory disease or relapse is mainly a result of leukemia cell resistance (3). Currently, the diagnostic stratification of ALL is based on a combination of clinical information and morphology, immunology, cytology, and molecular biology (MICM) and can be used for comprehensive prognostication. Meanwhile, early treatment response is also an independent predictor of prognosis in pediatric ALL. Poor treatment response, especially for patients with glucocorticoid resistance, often predicts poor prognosis (4). Several studies have found that certain molecular abnormalities are associated with a poor prognosis and drug resistance. For instance, *IKZF1* deletions are associated with tyrosine kinase inhibitor resistance in Philadelphia chromosome-positive (Ph+) leukemia (5). Furthermore, *CDKN2* deletions are associated with chemotherapy resistance in ALL (6). As for the mechanism of glucocorticoid resistance, more and more molecular abnormalities have been discovered, for example, *CREBBP* and *NT5C1* mutations have been reported to be associated with glucocorticoid resistance (7), however, there are still far more unknown. Therefore, a more detailed molecular understanding can help us screen high-risk patients at initial diagnosis, allowing early intervention and improvements in the cure rate among pediatric patients, and molecular information has become an important part of diagnosis and risk stratification. In this study, MICM typing and gene mutations were analyzed in ALL patients admitted to our center in order to identify the gene mutations associated with glucocorticoid resistance in ALL, and guild advancements in molecular diagnosis/risk stratification and drug target screening for ALL.

## Methods

### Clinical Data

This study was reviewed by the ethics committee of Guangdong Provincial People's Hospital and Guangdong Academy of Medical Sciences. Samples were collected after obtaining informed consent from the patients' guardians. A total of 71 children with ALL admitted to our center between September 2019 and September 2021 were enrolled. Bone marrow or peripheral blood samples collected at initial diagnosis were used for genetic testing *via* whole exome sequencing (WES). Meanwhile, fluorescence *in situ* hybridization (FISH), flow cytometry, karyotyping, RT-PCR analysis, routine laboratory tests, and physical examination were also performed. Treatment was started immediately upon diagnosis. The treatment schedule followed the SCCLG-ALL-2016 protocol^1^. This protocol was initiated with a prednisone test at a dose of 60 mg/m^2^·d for 7 consecutive days or >200 mg/m^2^ week. Prednisone sensitivity was determined according to the SCCLG-ALL-2016 protocol; that is, a primary naive lymphocyte count of <1 × 10^9^/L in peripheral blood at the end of the prednisone test was considered an indicator of sensitivity, whereas a value >1 × 10^9^/L was considered an indicator of resistance. Cerebrospinal fluid (CSF) was examined using cell smears and flow cytometric analysis. The diagnosis of central nervous system (CNS) leukemia was based on the SCCLG-ALL-2016 protocol.

### DNA Sequencing

Before treatment, 2 mL bone marrow or peripheral venous blood samples were obtained from the ALL patients. EDTA was added as an anticoagulant, and DNA was extracted from the collected samples. Whole exome gene sequencing was performed to screen gene mutations *via* the ALL DNA whole exome sequencing gene chip (Illumina) on an Illumina sequencer (Illumina Nextseq500, US). The STRING online software (https:www.string-db.org) was used to conduct signaling pathway analysis for all identified gene mutations.

### Follow-Up

Follow-up was conducted *via* hospital visits or telephone discussions. The follow-up continued until September 30, 2021. Overall Survival was defined as the duration between diagnosis and death or the final follow-up.

### Statistical Analysis

We used SPSS 13.0 software for statistical analysis. Comparisons of categorical variables were performed using Pearson's Chi-squared test or Fisher's exact test. OS was determined using Kaplan–Meier analysis. *P* < 0.05 was considered statistically significant.

## Results

### Clinical Features

A total of 71 children (age, 1–14 years) with ALL confirmed using bone marrow smears underwent MICM subtyping. Ten patients (14.1%) had T-cell ALL (T-ALL) and 61 (85.9%) had B-cell ALL (B-ALL). There were 43 boys and 28 girls, with a male to female ratio of 1.5:1. The ranges of white blood cell (WBC) counts and lactate dehydrogenase (LDH) levels were 0.98–413 × 10^9^/L and 101–16863 U/L, respectively. Karyotype analysis revealed a normal karyotype in 24 patients (33.8%) and abnormal karyotype in 36 patients (50.7%); however, no chromosomal information was obtained for 11 patients (15.4%). Fusion gene detection based on FISH and RT-PCR revealed no abnormalities in 47 patients (66.2%). However, 8 patients (11.2%) were *TEL*-*AML1*+, 5 (7.0%) were *BCR*-*ABL*+, 5 (7.0%) were *E2A*-*PBX1*+, 2 (2.8%) were *KMT2A* +, 2 (2.8%) were *HOX11*+, 1 (1.4%) was *TEL*-*ABL*+, and 1 (1.4%) had a *TEL* deletion. Seven patients (9.8%) were diagnosed with CNS leukemia (e.g., CNS2 and CNS3), including 3 with T-ALL, 4 with B-ALL, 1 with *BCR-ABL*+ disease, and 1 with *TEL-ABL*+ disease. All cases of relapse (7, 9.8%) involved B-cell bone marrow relapse; these cases included 1 case of *E2A-PBX1*+ disease, 1 of *HOX11*+ disease, 3 of *BCR-ABL*+ disease, and 1 of *TEL-AML1*+ disease (Table 1).

**Table 1:** Clinical feature of the 71 patients.

**Clinical features**	**Classification**	**Quantity**	**Percentage**
Subtyping		71	100%
	B	61	85.9%
	T	10	14.1%
Gender		71	100%
	Male	43	60.6%
	Female	28	39.4%
WBC		0.98–413 × 109/L	
LDH		101–16863 U/L	
Karyotype		71	100%
	Normal	24	33.8%
	Abnormal	36	50.7%
	None	11	15.5%
FISH or RT-PCR		71	100%
	Normal	47	66.2%
	*TEL*-*AML1*+	8	11.3%
	*BCR*-*ABL*+	5	7.0%
	*E2A*-*PBX1*+	5	7.0%
	*KMT2A*+	2	2.8%
	*HOX11*+	2	2.8%
	*TEL*-*ABL*+	1	1.4%
	*TEL* deletion	1	1.4%
CNS		7	9.8%
	*T*	3	4.2%
	*B* *BCR-ABL*+ *TEL*-*ABL*+	4 1 1	5.6%
Relapsed		7	9.8%
	*B* *BCR-ABL*+ *E2A*-*PBX1*+ *HOX11*+ *TEL*-*AML1*+	7 3 1 1 1	9.8%

### Gene Mutation and Signaling Pathway Analysis

**Figure 1:**
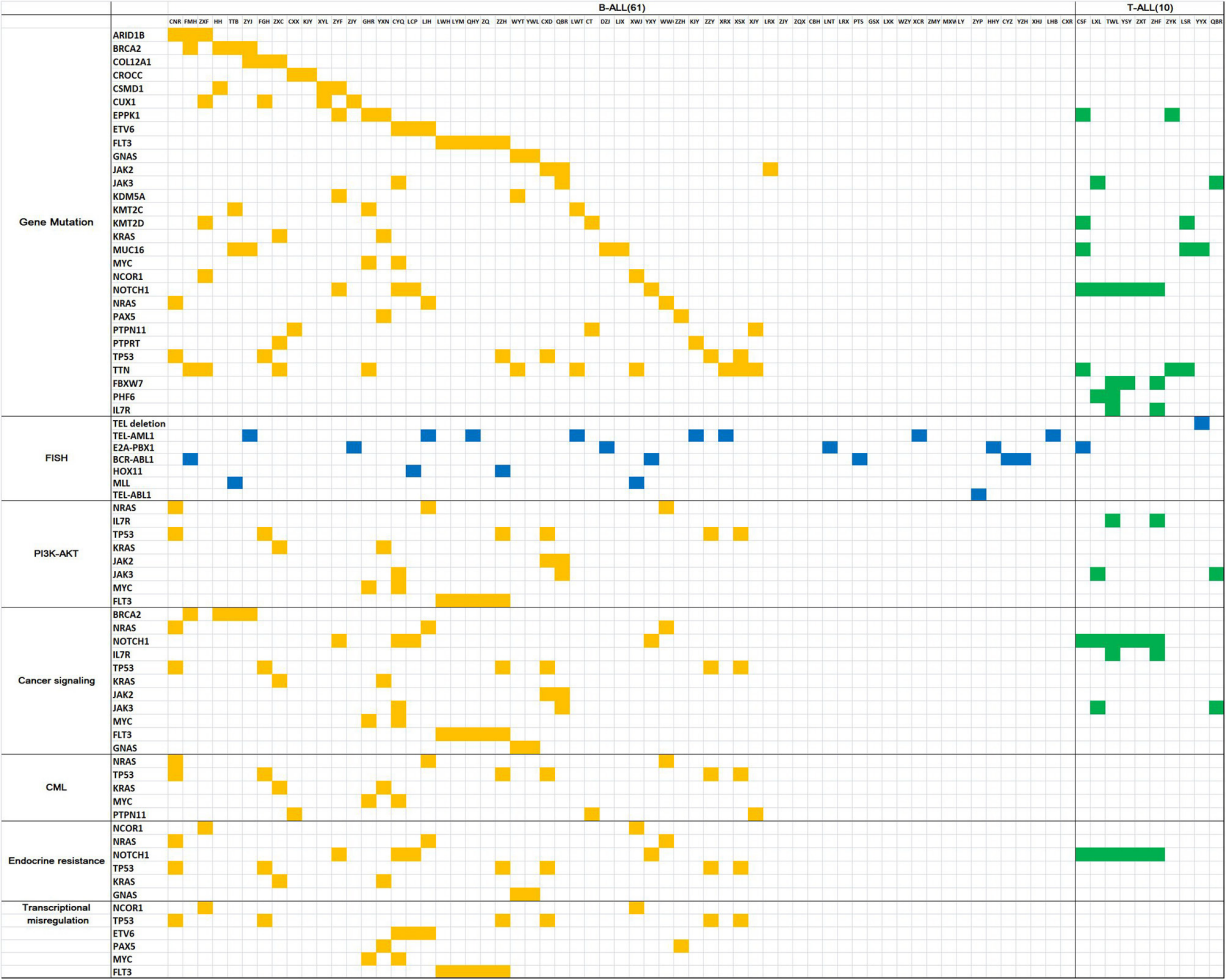
Gene mutation map for acute lymphoblastic leukemia. The horizontal columns represent the patient number, the vertical columns represent the gene name, the yellow squares represent positive gene mutation in B-ALL, the green squares represent positive gene mutationin T-ALL, the blue squares represent positive FISH results.

Gene mutation analysis revealed 10 genes with a high mutation frequency in B-ALL: *TTN* (24.4%), *FLT3* (14.6%), *TP53* (14.6%), *MUC16* (9.8%), *EPPK1* (9.8%), *NOTCH1* (9.8%), *CUX1* (9.8%), *BRCA2* (9.8%), *KMT2C* (7.3%), and *CSMD1* (7.3%). In T-ALL, the genes with a high mutation frequency were *NOTCH1* (54.5%), *FBXW7* (27.3%), *TTN* (27.3%), *MUC16* (27.3%), *PHF6* (18.2%), *KMT2D* (18.2%), *EPPK1* (18.2%), *FLT3* (18.2%), *IL7R* (18.2%), and *JAK3* (7.3%). Of the 7 children with CNS leukemia, 1 was *JAK2*+, 1 was *FLT3*+, and 1 was *MUC16*+ (Figure 1).

STRING analysis showed that the primary signaling pathways involved were cancer signaling (*BRCA2, NRAS, NOTCH1, IL7R, TP53, KRAS, JAK2, JAK3, MYC, FLT3*, and *GNAS*), cancer MIS transcription signaling (*NCOR1, TP53, ETV6, PAX5, MYC*, and *FLT3*), PI3K-Akt signaling (*NRAS, IL7R, TP53, KRAS, JAK2, JAK3, MYC*, and *FLT3*), endocrine resistance signaling (*NCOR1, NRAS, NOTCH1, TP53, KRAS*, and *GNAS*), and chronic myeloid leukemia signaling (*NRAS, TP53, KRAS, MYC*, and *PTPN11*) (Figure 1).

### Correlation of Gene Mutations With Prednisone Sensitivity

There were 13 children with *TTN*+ disease, accounting for 18.3% of all patients. Among them, 3 had T-ALL and 10 had B-ALL. One was *E2A-PBX1*+, 1 was KMT2A+, 2 were *TEL-AML1*+, and 1 was *BCR-ABL*+. The prednisone test revealed sensitivity in 6 children and resistance in 7 children (Table 2). Among the *TTN*- children, the prednisone test revealed sensitivity in 48 children and resistance in 10 children. The difference in prednisone sensitivity between *TTN*+ and *TTN*- children was analyzed using the Chi-square test, and a *P-*value <0.05 was obtained. Therefore, *TTN* mutations appeared to be associated with prednisone resistance (Table 4). There were 10 children with *NOTCH1*+ disease, accounting for 14.08% of all patients. Of them, 6 had T-ALL and 4 had B-ALL. Moreover, 1 was *E2A-PBX1*+ and 1 was *BCR-ABL*+. The prednisone test revealed resistance in 6 children and sensitivity in the other 4 (Table 3). Among *NOTCH1*- children, the prednisone test revealed sensitivity in 50 children and resistance in 11 children. The difference in prednisone sensitivity between *NOTCH1*+ and *NOTCH1*- children was analyzed using the Chi-square test, and a *P-*value <0.05 was obtained. Therefore, *NOTCH1* mutations appeared to be associated with prednisone resistance (Table 4).

**Table 2:** Clinical information from patients with *TTN*+ mutations.

**No**.	**Diagnosis**	**FISH**	**Chromosome**	WBC(^*^10^**9**^/L)	**LDH(U/L)**	**CNS**	Pred test^*^
1	ALL-B	BCR-ABL+	46,XX,t(9,22)(q34;q11)[9]/46,XX[1]	15.67	1,508	CNS2	R
2	ALL-B	None	46,XX[17]	4.18	182	Neg	S
3	ALL-B	TEL/AML1	46,XX[19]	6.03	863	Neg	S
4	ALL-B	TEL/AML1	46,XX[20]	7.07	323	Neg	S
5	ALL-B	MLL-AF9+	46,XY, add (11) (q?23),-13,+19[1]/46,XY[9]	2.19	480	Neg	S
6	ALL-B	None	46,XY,add(9)(?q24)[3]/47,XY,+21[2]/ 60–64,XXY,−2, −3, −6, −10, −13,+21,+mar1[cp3], −12, −14,+15,+16,-17,-18,+21[1]/46,XY[2]	7.58	459	Neg	S
7	ALL-B	None	47,XX,+21,inc.[1]	1.1	396	Neg	R
8	ALL-B	E2A-PBX1+	52,XY,t(1;19)(q23;p13), ?del(3)(p25),+5,+6,+8,+18,+20,+21[6]/46,XY[2];	47.6	1,783	Neg	R
9	ALL-B	None	None	3.29	450	Neg	S
10	ALL-B	c-MYC+	None	9.38	1,311	Neg	R
11	ALL-T	None	46,XY,*t*(1;21)(q41;q22)add(21)(q22)[2]/ 32–47,XY, −18, −19, −21,add(21)(q22)[cp4]/ 46,XY[2]	52.55	839	CNS2	R
12	ALL-T	None	46,XY[18]	16.08	830	CNS3	R
13	ALL-T	None	None	413	3,177	Neg	R

* *R, resistant; S, sensitive*.

**Table 3:** Clinical information from patients with *NOTCH1*+ mutations.

**No**.	**Diagnosis**	**FISH**	**Chromosome**	WBC(^*^10^**9**^/L)	**LDH(U/L)**	**CNS**	Pred test^*^
1	ALL-T	None	47,XX,+11[6]/46,XX[6]	132	5,100	Neg	R
2	ALL-T	None	46,XX[5]	47.65	480	Neg	R
3	ALL-T	None	46,XX,t(11;14)(p13;q11)[9]/ 46,XX[10],t(11;14)(p13;q11)	31.35	4,692	Neg	S
4	ALL-T	None	47,XY,+11[6]/46,XY[14]	56.67	2,395	Neg	S
5	ALL-T	None	46,XX[19]	70.38	3,484	Neg	S
6	ALL-T	None	45,XX,-21[4]/46,XX[16]	2.39	292	Neg	R
7	ALL-B	HOX11+	46,XX[20]	17.31	583	Neg	S
8	ALL-B	BCR-ABL1+	49-57,XX,+2,+5,+8,+9,t(9;22)(q34;q11), +17,+21,+der(22)t(9;22)(q34;q11), +der(22)t(1;22)(p31;q11),+mar1,+mar2,inc.[cp17] /46,XX[3]	11.12	421	Neg	R
9	ALL-B	E2A-PBX1+	52,XY,*t*(1;19)(q23;p13),?del(3)(p25), +5,+6,+8,+18,+20,+21[6]/46,XY[2]	47.6	1,783	Neg	R
10	ALL-B	None	46,XY[16]	22.35	341	Neg	R

* *R resistant, S sensitive*.

Table 4Clinical features associated with key gene mutations in pediatric acute lymphoblastic leukemia.

**Table:** 

	**TTN+**	**TTN-**	***P***	**NOTCH1+**	**NOTCH1-**	***P***	**FLT3+**	**FLT3-**	***P***
Age	4.78 ± 2.91	6.47 ± 3.99	0.038	7.70 ± 3.23	5.96 ± 3.89	0.266	6.20 ± 4.32	6.21 ± 3.82	0.8
Sex(F/M)	5(38.5%)/8(61.5%)	23(39.7%)/35(60.3%)	0.589	8(80.0%)/2(20.0%)	20(32.8%)/41(67.2%)	0.007	0(0.0%)/5(100.0%)	28(42.4%)/38(57.6%)	0.074
Subtype			0.263			0.000			0.457
B	10(76.9%)	51(87.9%)		4(40.0%)	57(93.4%)		5(100.0%)	56(84.8%)	
T	3(23.1%)	7(12.1%)		6(60.0%)	4(6.6%)		0(0.0%)	10(15.2%)	
WBC	45.05 ± 111.79	32.98 ± 39.03	0.707	43.88 ± 37.68	33.78 ± 61.50	0.617	12.59 ± 11.36	36.85 ± 60.44	0.33
LDH	969.30 ± 822.91	1,278.15 ± 2,410.58	0.651	1,957.10 ± 1,877.49	1,110.60 ± 2,240.91	0.258	457.83 ± 155.44	1,281.16 ± 2,274.48	0.38
Pred test			0.010			0.010			0.344
Pred resistant	7(53.8%)	10(17.2%)		6(60.0%)	11(18.0%)		2(40.0%)	15(22.7%)	
Pred sensitive	6(46.2%)	48(82.8%)		4(40.0%)	50(82%0.0)		3(60.0%)	51(77.3%)

**Table:** 

	**TP53+**	**TP53-**	***P***	**MUC16+**	**MUC16-**	***P***
Age		7.00 ± 4.56	6.13 ± 3.79	0.323	4.42 ± 2.14	6.40 ± 3.93	0.041
Sex(F/M)	3(50.0%)/3(50.0%)	25(38.5%)/40(61.5%)	0.444	2(28.6%)/5(71.4%)	26(40.6%)/38(59.4%)	0.426
Subtype			0.388			0.254
B	6(100.0%)	55(84.6%)		5(74.1%)	56(87.5%)	
T	0(0.0%)	10(15.4%)		2(28.6%)	8(12.5%)	
WBC	13.38 ± 10.20	37.52 ± 61.28	0.273	28.41 ± 18.77	35.62 ± 61.55	0.744
LDH	411.62 ± 157.55	1313.17 ± 2303.54	0.275	874.62 ± 779.29	1254.38 ± 2305.88	0.647
Pred test			0.556			0.054
Pred resistant	1(16.7%)	16(24.6%)		4(57.1%)	13(20.6%)	
Pred sesentive	5(83.3%)	49(75.4%)		3(42.9%)	50(79.4%)	

There were 5 children with *FLT3*+ disease, accounting for 7.04% of all patients; all of them had B-ALL, and one of them was *TEL-AML1*+. *FLT3* mutations did not appear to be associated with prednisone resistance (Chi-square test, *P-*value >0.05) (Table 4).

There were 6 children with *TP53*+ disease, accounting for 8.4% of all patients, and all of them had B-ALL without any special fusion genotype. *TP53* mutations did not appear to be associated with prednisone resistance (Chi-square test, *P-*value >0.05) (Table 4).

There were 7 children with *MUC16*+ disease, accounting for 9.8% of all patients. Of these patients, 2 had T-ALL and 5 had B-ALL. Moreover, 2 were *E2A-PBX1*+, 1 was *KMT2A* +, and 1 was *TEL-AML1*+. *MUC16* mutations did not appear to be associated with prednisone resistance (Chi-square test, *P-*value >0.05) (Table 4).

The relationship of *EPPK1, FBXW7*, and *PHF6* mutations with drug resistance could not be analyzed owing to the small number of cases.

### Correlation of Gene Mutations With Clinical Features and Survival

There were no statistically significant differences in leukocyte counts and LDH levels between patients with and without mutations in the following genes (Fisher's exact test, *P* > 0.05): *TTN, NOTCH1, FLT3, TP53*, and *MUC16*. In terms of age, the *TTN*+ group was younger than the *TTN*- group (Fisher's exact test, *P* < 0.05), and the *MUC16*+ group was younger than the *MUC16*- group (Fisher's exact test, *P* < 0.05). In terms of sex, there was a significantly higher proportion of female patients in the *NOTCH1*+ group than in the *NOTCH1*- group (Chi-square test, *P* < 0.05). In terms of disease type, the *NOTCH1*+ group had a significantly higher proportion of patients with T-ALL than did the *NOTCH1*- group (Chi-square test, *P* < 0.05) (Table 4).

**Figure 2:**
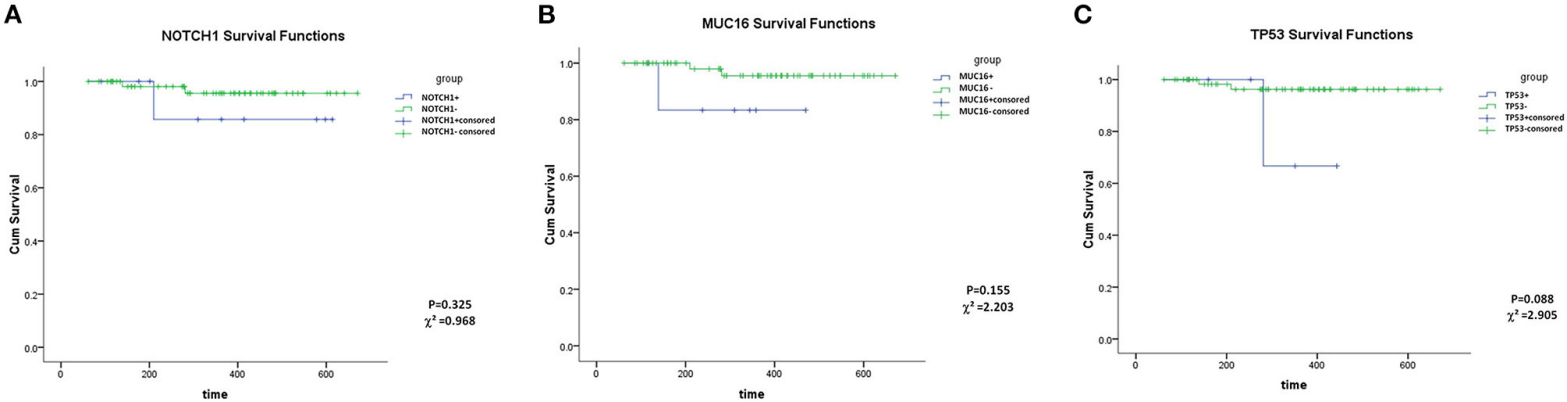
Overall survival analysis of NOTCH1 **(A)**, MUC16 **(B)**, TP53 **(C)** mutations.

*MUC16, TP53* and *NOTCH1* mutations showed no association with OS (Kaplan–Meier analysis, *P* > 0.05) (Figure 2). However, this may be a result of the short follow-up and the small number of cases. Groups of *TTN* and *FLT3* mutations could not be analyzed statistically owing to data limitations (Patients with TTN+ or FLT3+ were all alive at the final follow-up date).

## Discussion

Extraordinary advances have been made in the treatment of pediatric ALL, and prognosis has also improved significantly over the last few years. Nevertheless, more than 10% of cases of relapsed or refractory ALL cannot be cured (8). The cost of treatment has significantly increased, creating a burden for families and society at large. Given that the prognosis of pediatric ALL is associated with factors such as early treatment response, karyotype, fusion genes, gene mutations, and minimal residual disease (MRD), molecular diagnosis and risk stratification are important for the early identification of high-risk refractory ALL and the selection of appropriate therapy. Among the clinical features of ALL, an onset age of 1–10 years and a WBC count <50 × 10^9^/L are associated with a good prognosis. In contrast, an onset age of <1 year or >10 years and a WBC count >50 × 10^9^/L are associated with a poor prognosis. With respect to disease subtype, T-ALL is associated with a slightly worse prognosis than B-ALL (9). In terms of karyotype, it has been proven that hyperdiploidy as well as *t*_(12;21)_ (p13;q22) (*ETV6-RUNX1*) are associated with a good prognosis, whereas hypodiploidy, KMT2A rearrangements, and *Ph-like, IKZF1*, and *CRLF2* rearrangements are associated with a poor prognosis (10).

Early treatment response is an independent predictor of prognosis in pediatric ALL. The amount of MRD and prednisone sensitivity during induction therapy are important indicators of an early treatment response (11). Studies have shown that the risk of relapse and death is 3–5 times higher in children with MRD >0.01% at the end of induction therapy than in those with MRD <0.01% (12). Prednisone resistance is an independent prognostic factor for the risk-stratified diagnosis and treatment of ALL. Children with leukemia that is resistant to glucocorticoids are considered high risk for treatment failure (13). Glucocorticoids are effective treatment for ALL by leading to direct lymphocyte apoptosis (14). Studies have shown that glucocorticoids mainly induce apoptosis through *NR3C1* receptors on the surface of lymphocytes. Mutations in *NR3C1* as well as its co-stimulatory molecule *CREBBP* can lead to the transcriptional dysregulation of genes involved in glucocorticoid action, causing drug resistance (15). The increased expression of some anti-apoptotic molecules, including *BCL2, BCL-xL*, and *MCL1*, can also lead to decreased glucocorticoid sensitivity (16). Additionally, abnormalities in the bone marrow microenvironment are also associated with drug resistance. IL7 in the bone marrow microenvironment is believed to induce glucocorticoid resistance in T-ALL cells (17). In terms of signaling pathways, RAS/MEK/ERK, IL7R/JAK/STAT, and PI3K/AKT signaling have been found to be associated with glucocorticoid resistance. Currently, inhibitors targeting these signaling pathways, i.e., MEK inhibitors, AKT inhibitors, and JAK inhibitors, are being tested in clinical trials (18). Nevertheless, the mechanism underlying glucocorticoid resistance remains to be fully elucidated. Therefore, more clinical data as well as basic research are required to explore the mechanisms of drug resistance in ALL.

In this study, we analyzed clinical data, including clinical features, fusion genes, and gene mutation data, obtained from pediatric ALL patients treated at our center over the past 2 years. In terms of subtype distribution as well as sex, our findings were consistent with international and domestic data (19). Using FISH and RT-PCR results, we observed that *BCR-ABL* fusion occurred in 7% of our patients, which was slightly higher than the rate reported previously (3–5%). *TEL-AML1*fusion was observed in 11.2% of cases, which was lower than the previously reported rate of 25% (20). These differences may be related to the small number of cases in our study. [*HOX*11*t*_(10, 14)_] (20% in T-ALL), *E2A-PBX1* (7%), *KMT2A* (2.8%) and *TEL* deletions (1.8%) rates were similar to those reported previously (21–24). All cases of CNS involvement were detected at initial diagnosis or during induction therapy. The incidence of CNS leukemia in our study was 9.8%, higher than the rates of 3–8% reported in previous studies from China and the rest of the world (25, 26). This difference could be related to the small sample size in our study and inadequate sedation during lumbar puncture.

Gene mutation analysis revealed 10 genes with a high gene mutation frequency in T-ALL: *NOTCH1* (54.5%), *FBXW7* (27.3%), *TTN* (27.3%), *MUC16* (27.3%), *PHF6* (18.2%), *KMT2D* (18.2%), *EPPK1* (18.2%), *FLT3* (18.2%), *IL7R* (18.2%), and *JAK3* (7.3%).Of these genes„ the mutation frequency for *FLT3* mutations were slightly more frequent than previously reported (27–29), *MUC16*, which showed a high mutation frequency in our study, was less common in previous reports. Moreover, to our knowledge, our study is the first to detect *EPPK1* and *TTN* mutations in T-ALL. The top 10 genes with a high mutation frequency in B-ALL were *TTN* (24.4%), *FLT3* (14.6%), *TP53* (14.6%), *MUC16* (9.8%), *EPPK1* (9.8%), *NOTCH1* (9.8%), *CUX1* (9.8%), *BRCA2* (9.8%), *KMT2C* (7.3%), and *CSMD1* (7.3%). These findings were different from those of previous studies from outside China, which most commonly reported *PAX5* and *IKZF* mutations (30). Nevertheless, our results were consistent, to a certain extent, with previous reports from China. For instance, Zheng et al. reported that *FLT3* and *TP53* mutations are common in Chinese ALL patients (31), and Zhang et al. reported high rates of *FLT3* mutations in B-ALL (32). In contrast, a lower rate of *FLT3* mutations (4–5%) has been reported in international studies (33, 34). In our study, all *TP53* mutant chromosomes had a normal karyotype, although previous studies have shown that *TP53* mutations usually occurred in hypodiploidy. All these differences may be related to differences in ethnicities as well as the number of cases.]. *NOTCH1*mutations are typically observed in T-ALL. However, studies have shown that NOTCH signaling receptors are also present on the surface of B cells (35), and NOTCH signaling has been implicated in B-ALL resistance (36).In our study, *NOTCH1*mutations were detected in 9.8% of B-ALL cases, and all 4 *NOTCH1*+ B-ALL cases were in the high-risk group, suggesting that *NOTCH1* mutations may be associated with drug resistance and a poor prognosis in B-ALL.

The 5 genes with the highest mutation frequencies were analyzed for their correlation with prednisone sensitivity. *TTN* mutations as well as *NOTCH1* mutations were found to show a high frequency in both B-ALL and T-ALL. Both these mutations were associated with resistance in the prednisone test. To our knowledge, the association between *TTN* mutation and glucocorticoid resistance has not been reported previously. While some studies have explored the relationship between Notch1 signaling and glucocorticoid resistance in T-ALL (37–41). However, these studies were largely performed in cell lines or animal models. Further, in studies examining clinical samples, the sample size was very limited. Therefore, our *NOTCH1* mutation data are notable because our study is the first to report results from a large number of clinical samples. *CREBBP* and *NT5C1* mutations, which have previously been linked to glucocorticoid resistance, were not detected in our study. *TP53, MUC16*, and *FLT3* mutations showed no correlation with prednisone resistance, although statistical analysis revealed that *TTN* and *MUC16* mutations were associated with a lower age among pediatric ALL patients.

The *TTN* gene (Titin, myonectin) is located on chromosome 2 at 2q31.2 and encodes a striated muscle protein. *TTN* mutations are associated with neuromuscular disease, cardiomyopathy, and the development of solid tumors (42). In terms of drug resistance, *TTN* mutations are associated with insulin tolerance in metabolic disease (43, 44) and the degree of anthracycline-induced myocardial damage (45). Lips et al. found that *TTN* is associated with chemotherapeutic drug tolerance in breast cancer (46, 47). Further, Jia et al. found *TTN* mutations in various solid tumors, such as breast cancer, lung cancer, and cervical cancer, and showed that these mutations were correlated with treatment response and prognosis (48). However, reports of *TTN* mutations in hematological tumors are currently rare. Skoczen et al. reported a *TTN* mutation in 1 patient with ALL and Netherton syndrome (49). Hence, although *TTN* mutations may be associated with glucocorticoid resistance in ALL, further data and research are required to validate this finding. The frequency of *MUC16* mutations was also higher in the prednisone resistance group, although the difference was not statistically significant. The *MUC16* (Mucin16) gene encodes a protein belonging to the mucin family, which is thought to be involved in barrier formation, protecting epithelial cells from pathogens (50). The product of this gene has been used as a marker for different cancers, and its expression is associated with a poor prognosis (51, 52). However, this mutation has not been reported in ALL. Additionally, our data analysis also revealed that *NOTCH1* was associated with glucocorticoid resistance, consistent with previous reports of the link between mutations in this gene and a poor prognosis (53, 54).

## Conclusions

The clinical features and gene mutation profiles of 71 pediatric ALL patients were obtained and investigated in the present study. We analyzed the differences in clinical features between pediatric ALL cases from our center and those examined in previous studies and described the common gene mutations as well as signaling pathways involved in pediatric ALL. Of note, our study is the first to report the association between *TTN* and *NOTCH1* mutations and a low age of onset and glucocorticoid resistance in ALL. These findings could guide strategies for overcoming drug resistance and aid in the development of drug targets. However, owing to the small sample size of our study, more basic research and clinical studies examining a larger number of cases are required to validate our findings.

## Data Availability Statement

The original contributions presented in the study are publicly available. This data can be found here: NCBI Genebank, Accession ID PRJNA818462.

## Ethics Statement

The studies involving human participants were reviewed and approved by Ethics Committee Board of the Guangdong Provincial People's Hospital. Written informed consent to participate in this study was provided by the participants' legal guardian/next of kin.

## Author Contributions

JZ designed the study, carried out the statistical analysis and provided the data and pictures, drafted the initial manuscript, and approved the final manuscript as submitted. LZ and YW provided the chromosomal, FISH and RT-PCR information and approved the final manuscript as submitted. JP, BF, XL, and QY collected the clinical information for all the patients, and approved the final manuscript as submitted. All authors read and approved the final manuscript.

## Funding

This work was supported by funding from the Guangdong Basic and Applied Basic Research Foundation (Grant Number 2018A030313524).

## Conflict of Interest

The authors declare that the research was conducted in the absence of any commercial or financial relationships that could be construed as a potential conflict of interest.

## Publisher's Note

All claims expressed in this article are solely those of the authors and do not necessarily represent those of their affiliated organizations, or those of the publisher, the editors and the reviewers. Any product that may be evaluated in this article, or claim that may be made by its manufacturer, is not guaranteed or endorsed by the publisher.

## Footnotes

^1^ http://www.gdaca.org.cn/

## Abbreviations

ALL: Acute lymphoblastic leukemia
OS: Overall survival
CNS: Central nervous system
FISH: Fluorescence *in situ* hybridization
WBC: White blood cell
LDH: Lactate dehydrogenase
WES: Whole exome sequencing
MICM: Morphology, immunology, cytology and molecular biology
TKI: Tyrosine kinase inhibitor
MRD: Minimal residual disease
Ph+: Philadelphia chromosome-positive.
